# Advancing hybrid modeling of *Saccharomyces cerevisiae* fermentation with mixed carbon sources and urea in a mini-stirred tank reactor

**DOI:** 10.1007/s00449-025-03222-5

**Published:** 2025-08-23

**Authors:** Jhonatan Valencia-Velásquez, Hector Andres Yaker-Moreno, Alejandro Martínez-Guerrero, Francisco Ibáñez-Espinel, José Ricardo Pérez-Correa, Nelson H. Caicedo-Ortega

**Affiliations:** 1 Departamento de Ciencias Biológicas, Bioprocesos y Biotecnología, Facultad de Ingeniería, Diseño y Ciencias Aplicadas, Universidad ICESI, Cali, Colombia; 2 Centro BioInc. Universidad ICESI, Cali, Colombia; 3 Advanced Process Control and Data Sciences, Natural Resources, SGS Chile, Puerto Madero # 130, Pudahuel, Santiago, Chile; 4 Departamento de Ingeniería Química y de Bioprocesos, Facultad de Ingeniería, Pontificia Universidad Católica de Chile, Casilla 306 Correo 22, Santiago, Chile

**Keywords:** Hybrid modeling, LSTM networks, Global optimization, Yeast culture, Mixed sugars, Crabtree effect

## Abstract

*Saccharomyces cerevisiae* is indispensable to industrial fermentation; however, many existing models fail to adequately represent the metabolic complexity of its growth on mixed carbon sources in defined media. In this study, we introduce a novel hybrid modeling framework for the batch cultivation of *S. cerevisiae*, utilizing sucrose, glucose, and fructose as carbon sources, and urea as a nitrogen source. The model decisively captures critical phenomena under aerobic conditions, including the Crabtree effect, diauxic shifts, and sequential sugar utilization—critical areas frequently oversimplified in current models. By integrating mechanistic kinetics with data-driven enhancements, the hybrid model significantly improves predictive accuracy relative to the purely mechanistic baseline, reducing the average prediction error by a factor of 1.9 during training and 2.0 during testing. This framework enables detailed simulation of culture dynamics and was carefully designed for modular integration into digital twin platforms and automated control systems, aligning perfectly with Industry 4.0 biomanufacturing trends. Furthermore, the model’s validation under conditions pertinent to emerging bioeconomies, such as those in Latin America, underscores its industrial applicability. Overall, this work delivers a scalable and precise tool for optimizing yeast-based bioprocesses, carrying significant implications for defined media formulation, metabolic engineering, and digital fermentation technologies.

**Supplementary Information:**

The online version contains supplementary material available at 10.1007/s00449-025-03222-5.

## Introduction

Industrial yeast cultivation in Latin America commonly relies on agro-industrial byproducts rich in mixed sugars, primarily sucrose, glucose, and fructose [1, 2], with urea as a nitrogen source [3]. Despite the widespread use of these substrates, most studies on *S. cerevisiae* fermentation focus on single-carbon-source conditions, predominantly glucose [4–9]. While these studies have provided valuable insights into yeast metabolism and process optimization, they may not fully capture the complexity of mixed-sugar utilization and its metabolic implications. In addition, the role of urea as a cost-effective nitrogen source, widely used in large-scale bioprocesses, has not been extensively explored in comparative studies. A deeper understanding of how *S. cerevisiae* metabolizes mixed sugars and urea is crucial for improving fermentation performance [10–12], particularly in industrial settings, where sugarcane-derived feedstocks are commonly employed and in laboratory-scale synthetic media designed for reproducibility [13–15].

The cultivation of *S. cerevisiae* is a complex and dynamic bioprocess with significant industrial relevance, particularly in the food and biotechnology sectors [16–18]. Its global market is projected to reach USD 8.5 billion by 2029, reflecting its growing economic impact [19]. As industries increasingly adopt advanced manufacturing strategies, there has been a surge in interest in mathematical modeling and automation to optimize yeast-based processes [17]. These advancements have the potential to enhance productivity by up to 40% while reducing quality-related costs by 70% [20]. Despite these benefits, *S. cerevisiae* fermentation remains challenging due to intricate metabolic shifts, such as the Crabtree effect, diauxic transitions, and product inhibition, which directly affect substrate utilization and byproduct formation [7, 21]. Understanding and predicting these metabolic dynamics is critical for improving process control and operational efficiency [22].

Effectively capturing the complexities of *S. cerevisiae* fermentation requires a structured approach to process representation [7, 23]. Digital tools for process optimization, real-time monitoring, and control rely on mechanistic models grounded in first principles, which describe yeast metabolism by integrating metabolic kinetics, physicochemical constraints, and reactor conditions [4, 23–26]. While these models offer strong predictive capabilities, increasing complexity can hinder parameter estimation and identifiability, potentially reducing their real-world applicability [8, 27–30]. Achieving a balance between model accuracy and computational efficiency remains a key challenge [31], requiring strategies that enhance model reliability while ensuring feasibility in industrial and laboratory settings [23].

One of the primary gaps in existing models is the limited consideration of mixed sugar utilization in *S. cerevisiae* fermentations. Although many studies have successfully modeled yeast metabolism using glucose or glucose–fructose mixtures [4–8, 32–34], few address the simultaneous consumption of sucrose and its interactions with other sugars. This omission limits the model’s applicability in industrial contexts, where sugarcane-derived feedstocks are commonly used [1, 2], as well as in laboratory settings that rely on defined synthetic media for reproducibility [13, 15, 35]. To develop more accurate digital solutions for yeast bioprocessing, it is essential to incorporate mixed sugar metabolism and urea utilization, ensuring models reflect both industrial requirements and modern trends in media formulation.

Enhancing model robustness and scalability requires an integrated framework that decomposes the system into distinct submodels, each capturing specific biological and physicochemical aspects of yeast fermentation. This approach facilitates parameter estimation, model validation, and adaptation across different operational conditions [23, 25, 26]. Previous studies have demonstrated that partitioning large models into independently parameterized subcomponents significantly improves predictive accuracy while maintaining computational efficiency [23, 36]. In addition, systematic calibration techniques such as pre–post-regression analysis (PRA) provide a structured method for identifying key fitting parameters, preventing excessive model complexity, and ensuring that only identifiable parameters are retained [37–39]. Applications of PRA in *S. cerevisiae* cultivations have led to more accurate and parsimonious models, reinforcing their effectiveness in improving models’ performance [37, 40, 41].

Hybrid modeling approaches, particularly those integrating physics-informed neural networks (PINNs), provide a promising solution to the challenges of mechanistic modeling [42–47]. By embedding physical constraints within neural architectures, these models enhance predictive accuracy while maintaining interpretability, thereby overcoming the limitations associated with purely data-driven methods [48, 49]. Hybrid models have succeeded in various bioprocess applications, improving parameter estimation, reducing computational complexity, and capturing nonlinear metabolic interactions that are difficult to describe with first-principles models alone [50–55]. In addition, their ability to integrate experimental data and mechanistic knowledge enables more robust generalization across different operating conditions, facilitating their application in real-time optimization and control strategies [56–58]. PINN-based hybrid models have been successfully applied to *S. cerevisiae* fermentation with single substrates, demonstrating superior accuracy and robustness compared to traditional mechanistic models [40, 43, 48, 49].

This study introduces a hybrid modeling framework for *S. cerevisiae* cultivation under mixed-sugar conditions, where sucrose, glucose, and fructose serve as carbon sources, and urea acts as a nitrogen source. The approach combines a mechanistic submodel with a PINN-based hybrid component to enhance prediction accuracy and parameter identifiability. Here, we focus on the biological submodel, deferring its integration with physicochemical and reactor submodels to a subsequent publication. This hybrid platform aims to facilitate real-time monitoring, optimization, and control, bridging the gap between laboratory-scale synthetic media and industrial fermentation processes.

## Materials and methods

### General methodology for developing an accurate hybrid model for mixed-sugar fermentations

In industrial bioprocesses, yeast cultures often rely on complex carbon and nitrogen sources, particularly in regions, where agro-industrial byproducts serve as feedstocks. Commonly, *S. cerevisiae* is cultivated using sugarcane-derived substrates rich in sucrose, glucose, and fructose [59], with urea as a nitrogen source. While this composition provides cost-effective nutrient availability, it also introduces metabolic complexities that influence fermentation performance. The simultaneous utilization of multiple sugars leads to metabolic shifts, such as diauxic growth, changes in substrate uptake rates, and the Crabtree effect, which alter fermentation efficiency and product formation [7, 60]. Beyond carbon metabolism, nitrogen availability plays a fundamental role in regulating yeast growth, metabolic activity, and stress responses [61]. Urea, a readily assimilable nitrogen source, is hydrolyzed into ammonium and bicarbonate via urease activity, impacting intracellular pH and nitrogen catabolite repression pathways [3]. The balance between carbon and nitrogen assimilation directly affects biomass yield, metabolite production, and the activation of nitrogen-sensing regulatory networks, which modulate amino acid biosynthesis and enzymatic activity. In addition, nitrogen depletion or excess can trigger shifts in fermentation kinetics, influencing ethanol yield and byproduct accumulation. Understanding the interaction between mixed-sugar metabolism and nitrogen utilization is, therefore, essential for optimizing fermentation efficiency, minimizing resource waste, and enhancing process robustness under industrial conditions.

**Fig. 1:**
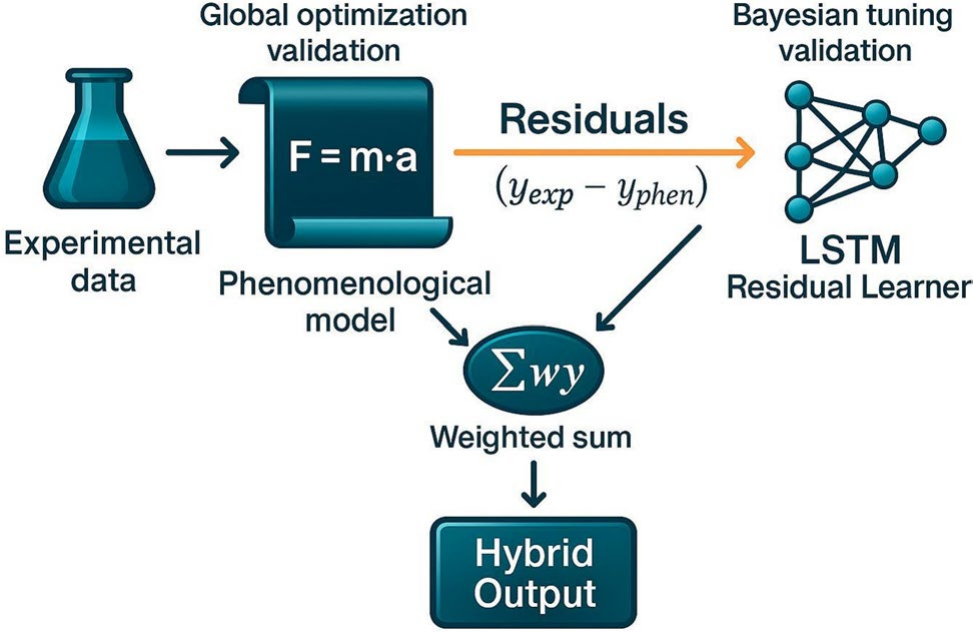
Sequential optimization workflow used in model calibration. (i) The phenomenological (first-principles) model is first calibrated by global optimization against the experimental data. (ii) Model–data discrepancies are expressed as residuals. (iii) An LSTM residual learner is subsequently trained on these residuals via Bayesian hyper-parameter tuning. (iv) Finally, the predictions from the calibrated phenomenological model and the LSTM are combined through a weighted sum operator to deliver the hybrid output, thereby exploiting both mechanistic insight and data-driven corrections

This study focuses on the development of a biological sub-model as a key component of an integrated hybrid framework for yeast fermentation. Given the complexity of microbial metabolism, constructing an accurate model requires robust methodologies that merge first-principles insight with data-driven learning [51, 55]. The following workflow (Fig. 1) details the methodology used to develop and validate the biological sub-model, ensuring a reliable representation of metabolic dynamics while maintaining computational efficiency.

1. Experiments: Three aerobic batch fermentations of *Saccharomyces cerevisiae* were performed (Exp 1–Exp 3), whose initial substrates, biomass loadings, and working volumes are summarized in Table 1. Exp 1 and Exp 2 were designed as biological replicates: identical bioreactors, media formulations, and operating conditions were used to capture intrinsic process variability while maximising statistical power for parameter estimation. The complete time-series from these two runs constituted the calibration (training) set. To challenge the model’s extrapolative capability, Exp 3 was conducted with deliberately shifted starting concentrations—most notably higher sucrose and biomass, and lower urea than the training experiments (Table 1)—and was *excluded* from any fitting procedure. Its trajectory served exclusively as an independent validation (test) set.
2. Data preparation and preprocessing: Due to the limited number of experimental sampling points, polynomial fitting was applied to the experimental data for sugars, urea, ethanol, and biomass concentrations. This approach maximized the coefficient of determination (*R*^*2*^) while ensuring consistency with process knowledge, allowing for the generation of additional reference points and capturing system behavior more effectively.
3. Parameter estimation via global optimization: To obtain a preliminary set of biologically meaningful parameter values, an initial calibration of the mechanistic model was performed using the Grey Wolf Optimization (GWO) algorithm in a Python environment [62]. This step provided parameter estimates close to their final values, facilitating convergence in subsequent refinements and reducing computational effort.
4. Pre–post-regression analysis: Following initial parameter estimation, PRA was conducted to evaluate parameter identifiability, sensitivity, and confidence intervals [37]. This analysis identified the parameters that significantly influenced the model output and determined which parameters could be fixed as constants, thereby enhancing model parsimony.
5. Parameter uncertainty analysis via Monte Carlo simulations: To assess the robustness of the model under varying conditions, Monte Carlo simulations were performed, generating a large number of random parameter sets to assess fitting stability. This analysis quantified the impact of parameter variability on model outputs and ensured that the model remained predictive across different fermentation conditions [40].
6. Model validation using independent experimental data: The model’s predictive accuracy was tested against an independent experimental data set with initial concentrations of sugars, biomass, and urea slightly different from those used for model calibration. This step ensured that the model could generalize across various operational conditions.
7. Neural network training on mechanistic model residuals: An LSTM network was trained to capture the residuals between the mechanistic model predictions and experimental data. This allowed the hybrid model to correct systematic discrepancies while retaining mechanistic interpretability.
8. Model uncertainty quantification: Monte Carlo simulations were conducted to evaluate the hybrid model’s generalizability.
9. Validation against independent experimental data: The final hybrid model was tested against an independent data set to assess its performance under real fermentation conditions. Validation metrics included prediction accuracy for biomass, sugar, ethanol, and urea consumption profiles.

**Table 1:** Initial conditions for the aerobic batch fermentations evaluated in this study

Exp	[Sucrose]_0_ (g/L)	[Glucose]_0_ (g/L)	[Fructose]_0_ (g/L)	[Biomass]_0_ (g/L)	[Urea]_0_ (g/L)
Train	32.7 ± 3.9	27.3 ± 3.8	27.1 ± 4.1	0.5 ± 0.1	2.6 ± 0.7
Test	39.6	27.5	25.8	1.2	2.3

The *Train* row shows the mean ± SD of two biological replicates (Exp 1 and Exp 2) that formed the calibration data set, whereas the *Test* row corresponds to the single independent validation batch (Exp 3)

### Biological submodel for describing aerobic batch cultivation of *S. cerevisiae*

The integrated model, based on a submodel framework [23], aims to address both micro-kinetics and transfer phenomena and macro-kinetics, including heat transfer, oxygen solubility, and mass transfer rate. It also incorporates operational conditions, such as stirring speed, air flow rate, and the geometric properties of *S. cerevisiae* culture, achieved through integrating submodels organized with constitutive equations and interconnected via parameters. This work focuses solely on the biological submodel. For its formulation, the following assumptions were considered:

1. For mass balance calculations, it is assumed that the culture density and rheology remain constant, given that high biomass concentrations were not reached during the process.
2. The pH and temperature of the culture are assumed to be constant.
3. It was assumed that no significant volume changes occurred due to evaporation. Under our operating conditions (30 °C, 0.8 VVM, ≤ 50 h), theoretical evaporation would account for approximately 6% (v/v) if no condensation occurred, based on the correlation proposed by Ask & Stocks (2022) [63]. Furthermore, the mini bioreactor system is equipped with a highly efficient condenser, allowing us to reasonably neglect volume losses due to evaporation.
4. The culture and biomass concentrations are assumed to be homogeneous within the stirred tank reactor, so a single sample represents the entire system.

The biological submodel comprises 6 state variables: biomass (X), ethanol (E), glucose (G), fructose (R), sucrose (S), and urea (U) in [gL^−1^]. This model was derived by applying mass balances, resulting in the differential equations system presented in Eqs. 1–6:

1 $$\frac{dX}{dt}=\frac{1}{V}\left[{r}_{X}V-X\frac{dV}{dt}\right]$$

2 $$\frac{dG}{dt}=\frac{1}{V}\left[{\left({F}_{i}{G}_{i}\right)}_{\left(t\right)}+V\left({r}_{gG}-{r}_{G}\right)-G\frac{dV}{dt}\right]$$

3 $$\frac{dR}{dt}=\frac{1}{V}\left[{\left({F}_{i}{R}_{i}\right)}_{\left(t\right)}+V\left({r}_{{g}_{R}}-{r}_{R}\right)-R\frac{dV}{dt}\right]$$

4 $$\frac{dS}{dt}=\frac{1}{V}\left[{\left({F}_{i}{S}_{i}\right)}_{\left(t\right)}-V{r}_{S}-S\frac{dV}{dt}\right]$$

5 $$\frac{dU}{dt}=\frac{1}{V}\left[{\left({F}_{i}{U}_{i}\right)}_{\left(t\right)}-V{r}_{U}+U\frac{dV}{dt}\right]$$

6 $$\frac{dEt}{dt}=\frac{1}{V}\left[V\left({r}_{Et}-{r}_{{c}_{Et}}\right)-Et\frac{dV}{dt}\right]$$

The glucose and fructose consumption rates (${r}_{G}$, ${r}_{R}$, respectively) depend on the amount of each substrate used for maintenance, ethanol production, and cell growth [64, 65], as shown in Eqs. 7 and 8. Equations 9–15 express the reaction rates for sucrose uptake rate [66, 67], yeast biomass growth rate, glucose production rate due to sucrose hydrolysis, fructose production rate, urea uptake rate, ethanol production rate, and ethanol uptake rate (gL^−1^ h^−1^), named ${r}_{S}$, ${r}_{X}$, ${r}_{{g}_{G}}$, ${r}_{{g}_{R}}$, ${r}_{Ur}$, ${r}_{Et}$, and ${r}_{{c}_{Et}}$, respectively. Equations 7, 8, 13, and 14 include a modified Leaky ReLU function to deactivate each rate function when certain substrates or products deplete. In this case, the Leaky ReLU function is normalized over the same term it evaluates; thus, the entire term oscillates between 0 and 1 without discontinuities [68]. These terms facilitate symbolic derivative calculation for solving the ODE system by eliminating indeterminacy in the denominator, improving the computational efficiency of the sensitivity calculations required by PRA:

7 $${r}_{G}=\left(({Y}_{G/X }*{\mu }_{G}*X)+\left({m}_{G}*X\right)+\left(\frac{{\mu }_{X}*X*{Y}_\frac{Et}{X}}{{Y}_\frac{Et}{G}}\right)\right)*\frac{max\left(\gamma *\left(G-0.001\right),\left(G-0.001\right)\right) }{\sqrt{{\left(G-0.001\right)}^{2}+\epsilon }}$$

8 $${r}_{R}=(({Y}_{R/X }*{\mu }_{R}*X)+\left({m}_{R}*X\right)+\left(\frac{{\mu }_{X}*X*{Y}_\frac{Et}{X}}{{Y}_\frac{Et}{G}}\right)*\frac{max\left(\gamma *\left(R-0.001\right),\left(R-0.001\right)\right) }{\sqrt{{\left(R-0.001\right)}^{2}+\epsilon }}$$

9 $${r}_{X}= {\mu }_{X}*X$$

10 $${r}_{S}={\alpha }_{S}\frac{S}{S+{K}_{S}}X$$

11 $${r}_{gG}={r}_{S}*{Y}_\frac{AF}{S}^{.}$$

12 $${r}_{gR}={r}_{S}*{Y}_\frac{AF}{S}^{.}$$

13 $${r}_{Ur}=\frac{{\mu }_{X}}{{Y}_\frac{X}{Ur}}*X*\frac{max\left(\gamma *\left(Ur-0.001\right),\left(Ur-0.001\right)\right) }{\sqrt{{\left(Ur-0.001\right)}^{2}+\epsilon }}$$

14 $${r}_{Et}=\left({Y}_{Et,X}*{\mu }_{X}*X\right)*\frac{max\left(\gamma *\left(R-0.001\right),\left(R-0.001\right)\right) }{\sqrt{{\left(R-0.001\right)}^{2}+\epsilon }}$$

15 $${r}_{Et,c}=\left({Y}_{c,Et/X}*{\mu }_{Et}*X\right)$$

The yeast biomass growth rate is influenced by the availability of simple sugars and ethanol concentration [7, 69, 70]. These dependencies are accounted for using Monod-type equations for each simple sugar, as shown in Eqs. 16 and 17. Glucose and fructose concentrations appear as affinity terms in these equations, while ethanol concentration is incorporated as an inhibitory factor. To account for glucose catabolic repression, adjustments were made to the maximum specific growth rate and the affinity constant for fructose, mediated by a modified Leaky ReLU function, as shown in Eqs. 18 and 19:

16 $${\mu }_{G}={\mu }_{max,G}\left(\frac{G}{{K}_{G}+G}\right)\left(\frac{{K}_{Et}^{Inh}}{{K}_{Et}^{Inh}+Et}\right)$$

17 $${\mu }_{R}={\mu }_{max,R}\left(\frac{R}{{K}_{R}+R}\right)\left(\frac{{K}_{Et}^{Inh}}{{K}_{Et}^{Inh}+Et}\right)$$

18 $${\mu }_{max,R}={\mu }_{max,R}+\frac{max\left(\gamma *\left(0.001-G\right),\left(0.001-G\right)\right) }{\sqrt{{\left(0.001-G\right)}^{2}+\epsilon } }*{({\mu }_{max,postG}-\mu }_{max,R})$$

19 $${K}_{R}={K}_{R}+\frac{max\left(\gamma *\left(0.001-G\right),\left(0.001-G\right)\right) }{\sqrt{{\left(0.001-G\right)}^{2}+\epsilon } }*{({K}_{R,postG}-{K}_{R}})$$

20 $${\mu }_{Et}={\mu }_{max,Et}\left(\frac{Et}{{K}_{Et}+Et}\right)\left(\frac{{K}_{Et,AF}^{Inh}}{{K}_{Et,AF}^{Inh}+AF}\right)$$

21 $${\mu }_{X}=\left( \left({\mu }_{G}+{\mu }_{R}\right)+{\mu }_{Et} \right)$$

When the sugars are fully depleted, the metabolism shifts to using ethanol as a carbon source through respiration [7, 71]. As shown in Eq. 20, the ethanol consumption term remains inactive when glucose or fructose is available due to an inhibition term similar to the one proposed by González-Hernández et al. [7]. Equation 21 demonstrates how the specific growth rate ${\mu }_{X}$ accounts for the contribution of growth in each substrate, as well as the effect of temperature, ${F}_{T}$ [72]. The general ODE system was solved with the DOP853 method from the library Scipy [73, 74], with absolute and relative errors of 1E-6 and 1E-6, respectively.

#### Data preparation and preprocessing

The fitting data set was generated by applying polynomial regressions to the experimental data for biomass, sucrose, glucose, fructose, urea, and ethanol concentrations. The LinearRegression function from *Sklearn* was used to fit polynomials that maximize the coefficient of determination (R^2^) while ensuring that the results remained consistent with established process knowledge. To avoid bias in the relevance analysis due to insufficient experimental points, 10 observations per parameter in the model were used, assuming that *S. cerevisiae* tendencies do not undergo sudden variations that could be ignored with the polynomial regression.

#### Parameter estimation via global optimization

Initial parameter estimation and subsequent submodel parameterization were conducted using a multivariate approach with the Grey Wolf Optimization (GWO) meta-heuristic algorithm. This algorithm simulates the hunting behavior of grey wolf packs to achieve convergence through a semi-randomized vector-based search procedure [62]. The normalized sum of the Mean Squared Errors (MSE) was used as the objective function (Eq. 22); this considers the weighted sum of squared normalized residual errors between the experimental mean and model predictions in each scenario. MSE is a suitable metric for model errors, as it assigns greater weight to larger deviations, effectively penalizing significant deviations more strongly than other statistical metrics, such as mean absolute error, while avoiding the potential bias introduced by the square root transformation in Root Mean Squared Error, which introduces a nonlinearity [75]. To account for differences in scale among state variables, squared errors were normalized by the maximum value of each corresponding experimental variable:

22 $$J(\theta )=min\left({\sum }_{j=1}^{N}{\sum }_{i=1}^{n}{\left(\frac{{x}_{i,j}^{model}-{\underline{x}}_{i,j}^{exp}}{\left({\underline{x}}_{i,j}^{exp}\right) }\right)}^{2}\right)$$

The initial parameter estimates were taken from values reported in the literature for *S. cerevisiae* cultures grown on glucose. Due to the lack of specific information about fructose, which led to using glucose-based bounds for fructose, an iterative bounding approach was implemented. Initially, a ± 30% bound around the initial values of each parameter was set for 20 epochs, if a parameter reached either the upper or lower limit during estimation, the bound was recalibrated to ± 10% around the current estimated value while checking that each recalculated parameter bound was not more than an order of magnitude away from the literature-based parameter bounds. This process was repeated for a total of 160 epochs, where the GWO algorithm reported no improvement in the global optimum and no further bound recalculation. The literature-based parameter bounds and corresponding references are presented in Table 2.

**Table 2:** Selected parameter bounds for each term of the model

Parameters	Lower bound	Upper bound	Reference	Parameter	Lower bound	Upper bound	Reference
${\mu }_{maxEt}$	0.1	0.3	[7, 40]	${K}_{R}$	0.001	0.5	[7, 40, 76]^a^
${\mu }_{maxG}$	0.1	0.5	[7, 77]	${K}_{R,postG}$	0	0.5	NA
${\mu }_{maxR}$	0.05	0.5	[7]^a^ [77]	${K}_{S}$	0.1	20	NA
${\mu }_{maxR,F,postG}$	0.04	1.5	NA	${Y}_\frac{AF}{S}^{dis}$	0.4	0.6	[7]
${m}_{G}$	0.01	0.09	[40]^a^	${Y}_\frac{Et}{G}$	0.3	0.8	[37, 76]^a^ [77]
${m}_{R}$	0.01	0.09	[40]^a^	${Y}_\frac{Et}{R}$	0.3	0.8	[37, 76]^a^
$\alpha$	4	30	This work	${Y}_\frac{Et}{X}$	10	20	[40, 76]
${K}_{in{h}_{AF}}$	0.01	0.2	This work, [78]	${Y}_{\frac{Et}{X},c}$	5	50	This work, [7]
${K}_{Et}$	0.01	0.15	[7, 40, 76]	${Y}_\frac{G}{X}$	10	50	[7, 40]^a^
${K}_{i,Et}$	15	30	[7, 40]	${Y}_\frac{R}{X}$	10	50	[7, 40]^a^
${K}_{G}$	0.001	0.5	[7]^a^	${Y}_\frac{X}{Ur}$	3	5	[7]

“This work” refers to the bounds that were calculated based on experimental data reported in this article. “NA” refers to parameters that were not found in the literature and could not able to be calculated from experimental data

^a^Some fructose-related parameters were estimated considering glucose kinetics from the references

The parameterization method was based on the OriginalGWO function from the *mealpy* library [79], and the polynomial regressions for the data sets were developed with the function LinearRegression by the *scikit-learn* library [80].

#### Pre–post-regression analysis

Pre–post-regression analysis (PRA) based on local parameter sensitivity is a standard approach for assessing model practical identifiability by evaluating different parameter sets (θ). To calculate the metrics (identifiability, sensitivity, and confidence intervals), the algorithm first computes the absolute sensitivity of the model response to parameter variations [37, 38, 81]. This computation is performed through a symbolic differential solution around an initial parameter set in this case, the parameters estimated by the GWO method.

Each metric has specific selection criteria:

1. Significance: A parameter’s *t* value must be greater than 2, indicating that the parameter is significantly different from zero and thus contributes to the model.
2. Identifiability: This condition confirms that a parameter can be reliably estimated from the model’s experimental data and that its value accurately represents the mathematical term, where it appears. If two parameters are correlated, the same model response can be obtained through different combinations of their values, which may lead to inconsistencies under stoichiometric or mass balance principles [28, 29]. For a parameter to be considered identifiable, it must satisfy $\left|{\rm K}_{ik}\right|<0.95$.
3. Sensitivity: While there is no universal numerical threshold for sensitivity, ${G}_{j}^{prom}$ it should be nonzero for at least one variable. It should also not be orders of magnitude smaller than the maximum ${G}_{j}^{prom}$ of each variable across all simultaneous evaluations. This condition indicates that changes in the parameter have a meaningful impact on the model’s response. In practice, sensitivity is often evaluated using a graphical analysis of the resulting values.

To apply these criteria, each metric is checked in sequence in the mechanistic model. Parameters that fail to meet a criterion are fixed before moving on to the next metric. First, all nonsignificant parameters are fixed, followed by all non-identifiable parameters, and then the sensitivity of each remaining parameter is examined. The sensitivity differential equations are solved using the CVODES solver through the CasADi library [82], which symbolically represents the system’s Jacobian and Hessian matrices. This approach helps prevent numerical instability that can arise from parameter oscillations [83].

#### Monte Carlo simulations

Parameter uncertainty was evaluated using Monte Carlo simulations with 100 iterations (*N* = 100). Each phenomenological parameter value was varied independently, following a normal distribution with a 5% standard deviation. This step helps quantify variability, providing a robust measure of both models’ predictive capabilities across different conditions.

#### Model validation

To evaluate the predictive capacity of the mechanistic and hybrid models, an independent test data set, distinct from the training data sets, was used. This test data corresponds to an experiment with initial conditions that slightly differ from those used in the model fitting, as detailed in Table 1.

#### Phenomenological models with neural networks: formulation, architecture, and training

**Fig. 2:**
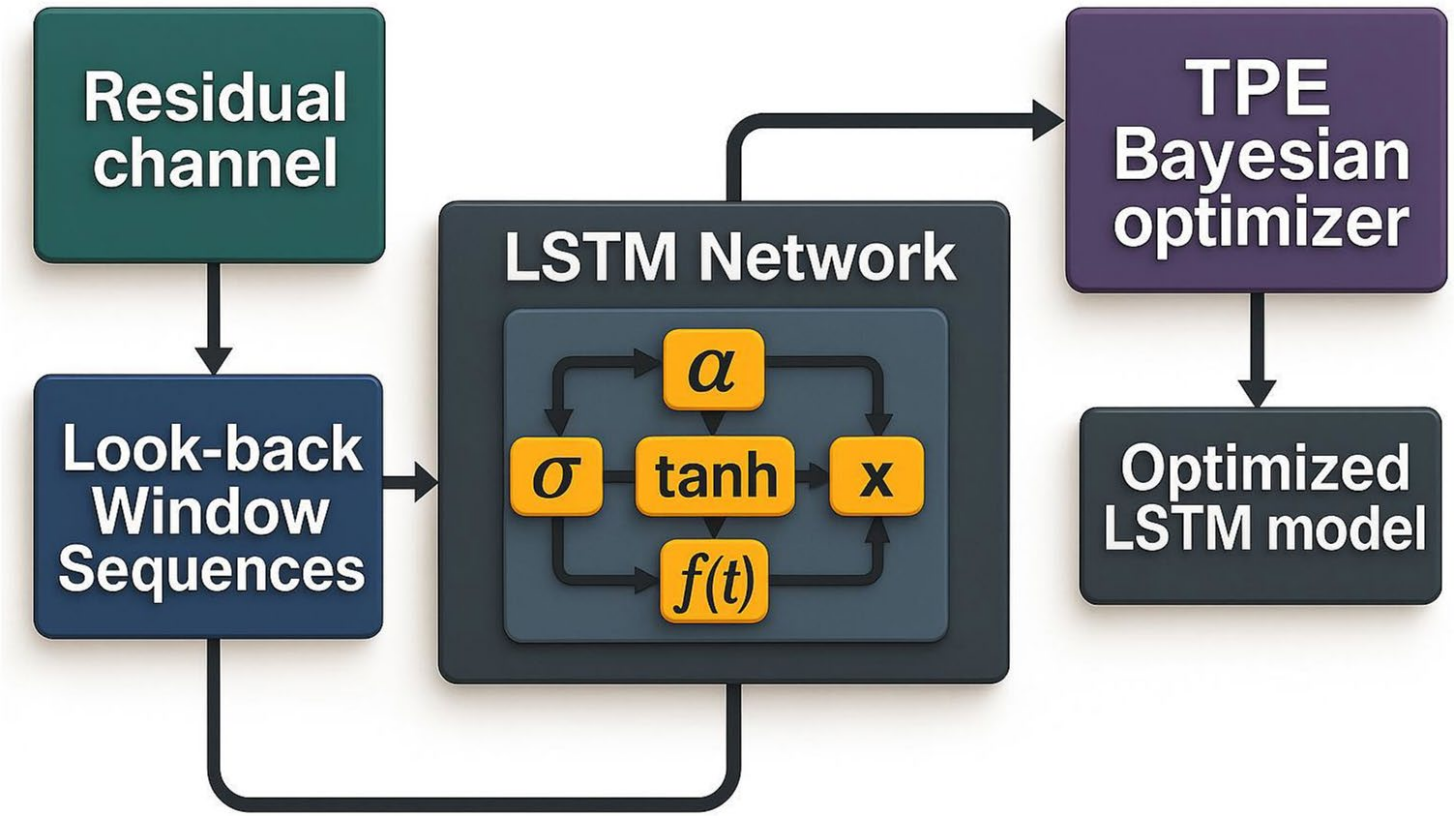
Schematic workflow for LSTM hyper-parameter optimization. Normalized residual sequences are transformed into look-back window inputs for the LSTM network. The resulting model undergoes hyper-parameter tuning via a Tree–Parzen estimator (TPE) Bayesian optimizer, yielding an optimized LSTM model structure for residual forecasting

Phenomenological (first-principles) models encode mass-balance and kinetic constraints, yet they often under-represent the high-order, state-dependent nonlinearities that emerge in yeast metabolism under industrial conditions. To correct these systematic deficiencies, a residual-learning strategy is adopted, whereby a long–short-term memory (LSTM) network is trained exclusively on the error time-series generated by the mechanistic model. Figure 1 summarizes the overall workflow, whereas Fig. 2 details the internal LSTM architecture and its bidirectional coupling with the phenomenological core. The present subsection now provides an explicit, step-by-step technical description of the LSTM construction and its integration within this hybrid framework.

1. Residual generation and normalization

For each experimental trajectory, we first simulate the mechanistic model using the parameters obtained in Sect.  2.3.2. The pointwise residual is defined as

$$r\left( {ti} \right) = y\exp \left( {ti} \right) - yph\left( {ti} \right),i = 1, \ldots ,N.$$

Because the magnitudes of r(t) differ across state variables, each residual channel is scaled to [0, 1] using a feature‑wise min–max transform. The resulting matrix R ∈ ℝ^{N × m} (with *m* = 6 for biomass, glucose, fructose, sucrose, urea, and ethanol) constitutes the only input to the data‑driven stage. Scaling guarantees numerical stability of the subsequent gradient‑based optimization without altering the physical meaning of the error dynamics.

2. Sequence construction for temporal learning

Temporal context is captured with a fixed look‑back window of length *p* = 5. For each time index i we extract the ordered tensor:

$$X^{{(i)}} = [r(t_{i} ),r(t_{{i - 1}} ), \ldots ,r(t_{{ip + 1}} )] \in \mathbb{R}^{{p \times m}} ,y^{{(i)}} = r(t_{{i + 1}} )$$

,so that the network is trained to predict the next residual given the last p observations. Inside each LSTM cell, the computation unfolds as follows:

- Input gate: *i*_*k* = *σ*(*W*_*i*·[*h*_{*k*-1}, *x*_*k*] + *b*_*i*)—decides how much of the new residual observation *x*_*k* enters the cell state.
- Forget gate: *f*_*k* = *σ*(*W*_*f*·[*h*_{*k*−1}, x_*k*] + *b*_*f*)—scales the previous cell memory c_{*k*−1}, enabling the network to discard outdated error patterns.
- Candidate cell: *ĉ*_*k* = tanh(W_c·[h_{* k*−1}, *x*_*k*] + *b*_*c*)—proposes a non‑linear transformation of x_k.
- Cell update: *c*_*k* = *f*_*k* ⊙ c_{*k*−1} + *i*_*k* ⊙ *ĉ*_*k*—blends old memory with the new candidate, filtered by the gates.
- Output gate: *o*_*k* = *σ*(*W*_o·[*h*_{*k*−1}, *x*_*k*] + *b*_*o*)—determines how much of the updated memory is exposed.
- Hidden state: *h*_*k* = *o*_*k* ⊙ tanh(*c*_*k*)—passed to the next time step (or next layer) and ultimately to the dense output layer.

This gated architecture solves the vanishing‑gradient problem inherent to long sequences and allows the model to retain long‑range correlations in the mechanistic residuals—for instance, systematic lagged errors caused by biomass‑dependent yield shifts or slow substrate‑inhibition kinetics. Because the cell state c carries additive updates (*f*_*k* ⊙ c_{*k*−1}), the LSTM effectively integrates relevant error patterns over time, providing a dynamic correction term that respects both short‑term oscillations (captured by *i*_*k*) and slow drifts (captured by *c*_*k*).

The final hidden state *h*_*p*—summarizing the entire p‑step history—is fed to a linear dense layer of size m, which outputs *ŕ*(*t*_{*i* + 1}). Training, therefore, minimizes the one‑step residual loss but, thanks to the LSTM’s recurrent nature, the network implicitly learns higher‑order multi‑step mappings, which manifests in improved open‑loop forecasts.

3. Bayesian hyper‑parameter optimization

The two calibration batches (Exp 1 + Exp 2) were first partitioned into an 80% fitting subset and a 20% internal validation subset. Only the validation subset was consulted when computing the loss used by the optimizer and for triggering early stopping. Hyper-parameters were tuned with a tree-structured Parzen estimator (TPE) over 100 iterations, exploring:

- Neuron units ∈ {32, 64, 128, 256} (per LSTM layer)
- Layers = 2
- Activation ∈ {ReLU, ELU, tanh, sigmoid, Leaky ReLU(α ∈ [0.01, 0.3])}
- Dropout, recurrent_dropout ∈ [0.0, 0.5]
- Learning‑rate ∈ 10^{(− 5, − 2)}
- Batch‑size ∈ {16, 32, 64, 128}
- Optimizer ∈ {Adam, RMSprop, Nadam}

The validation loss (Eq. 23) was minimized across these trials. The best-scoring configuration was then re-trained on the full 80% fitting subset with early stopping (patience = 15 epochs) before its performance was assessed against the independent test batch (Exp 3):

23 $$MS{E}_{val}=\frac{1}{M}{\sum }_{j=1}^{M}{\left({\widehat{y}}_{j}-{y}_{j}\right)}^{2}$$

4. Hybrid coupling and weighted sum

The hybrid framework linearly combines the phenomenological model predictions with the LSTM outputs:

24 $${y}_{hybrid}\left(t\right)={\lambda }_{pheno}*{y}_{pheno}\left(t\right)+{\lambda }_{ml}*{y}_{ml}\left(t\right)$$

Equation 24 combines the outputs of the phenomenological model (${y}_{pheno}$) and the machine learning model (${y}_{ml}$). The terms ${\lambda }_{pheno}$ and ${\lambda }_{ml}$ represent weighting factors assigned to each model to balance performance between the training and validation data sets. These weights were set to 0.3 and 0.7, respectively, by trial and error.

### Experimental setup for aerobic batch fermentations

An industrial baker’s-yeast strain, *Saccharomyces cerevisiae* ELB3 (courtesy of a national commercial yeast producer and maintained in the Biochemical Engineering culture collection, Universidad ICESI), was precultured for 16 h at 30 °C in YMG medium (10 g L⁻^1^ glucose, 3 g L⁻^1^ yeast extract, 3 g L⁻^1^ malt extract, pH 5.5). The resulting biomass served as inoculum for batch fermentations conducted in 500 mL MiniBioBundle microbial bioreactors equipped with my-Control units and Peltier-cooled condensers (Applikon^®^, Delft, The Netherlands). Experiments were conducted using working volumes of 300 and 400 mL. The fermentation broth contained (g L⁻^1^): H₃PO₄ (1.4), MgSO₄·7H₂O (0.44), MnSO₄ (0.069), pantothenate (vitamin B5, 0.012), thiamine (vitamin B1, 0.012), pyridoxine (vitamin B6, 0.006), biotin (vitamin B7, 0.0006), and various initial concentrations of sucrose, glucose, fructose, urea, and biomass as listed in Table 1. Vitamins were sterilized by filtration through a 0.22 μm nylon filter, whereas sugars, urea, and salts were autoclaved separately at 121 °C for 20 min.

The experiments were performed under aerobic conditions using a batch strategy. This approach allowed for the identification of distinct phases in which the cells predominantly exhibit specific metabolic states. In addition, kinetic phenomena—such as the Crabtree effect, diauxic shift, and product inhibition—were more pronounced in these cultures compared to fed-batch or anaerobic systems. All fermentations were maintained at a controlled temperature of 30 °C. A continuous airflow was provided at a constant rate of 0.8 VVM, and the agitation speed was set to 308 rpm. The initial pH was around 5.0 and was maintained between 4.0 and 5.0 throughout the process. Temperature, pH, and dissolved oxygen levels were monitored online using the Applikon^®^ system, while the exhaust gas composition was continuously measured with O₂ and CO₂ sensors (BlueSens^®^, Germany).

### Analytical methods

The biomass concentration was measured offline by a spectrophotometric method (UV–Vis—Thermo Scientific—480–300000 Genesys 15), which correlates with dry weight. The concentrations of sucrose, glucose, fructose, and ethanol in the supernatants were determined by liquid chromatography (UHPLC), (Thermofisher Scientific, UltiMate 3000, USA), employing a chromatograph equipped with a refractive index detector and an Aminex^®^ HPX-87-H column (300 × 7.8 mm, 10 μm, TF) operated at 35 °C. The eluent used was a sulphuric acid solution of 5 mM at a flow rate of 0.6 mL min^−1^. Solutions of sucrose, glucose, fructose, and ethanol at concentrations between 0.1 and 8.0 g L^−1^ were used as standards. Urea concentration was determined using Megazyme’s Urea/Ammonia (Rapid) Assay Kit for each supernatant sample. More detail is provided in supporting information S1 and S2.

### Human and animal rights

No human or animal subjects were involved in this research.

## Results

### Mechanistic model performance in multi-substrate *S. cerevisiae* fermentation

**Fig. 3:**
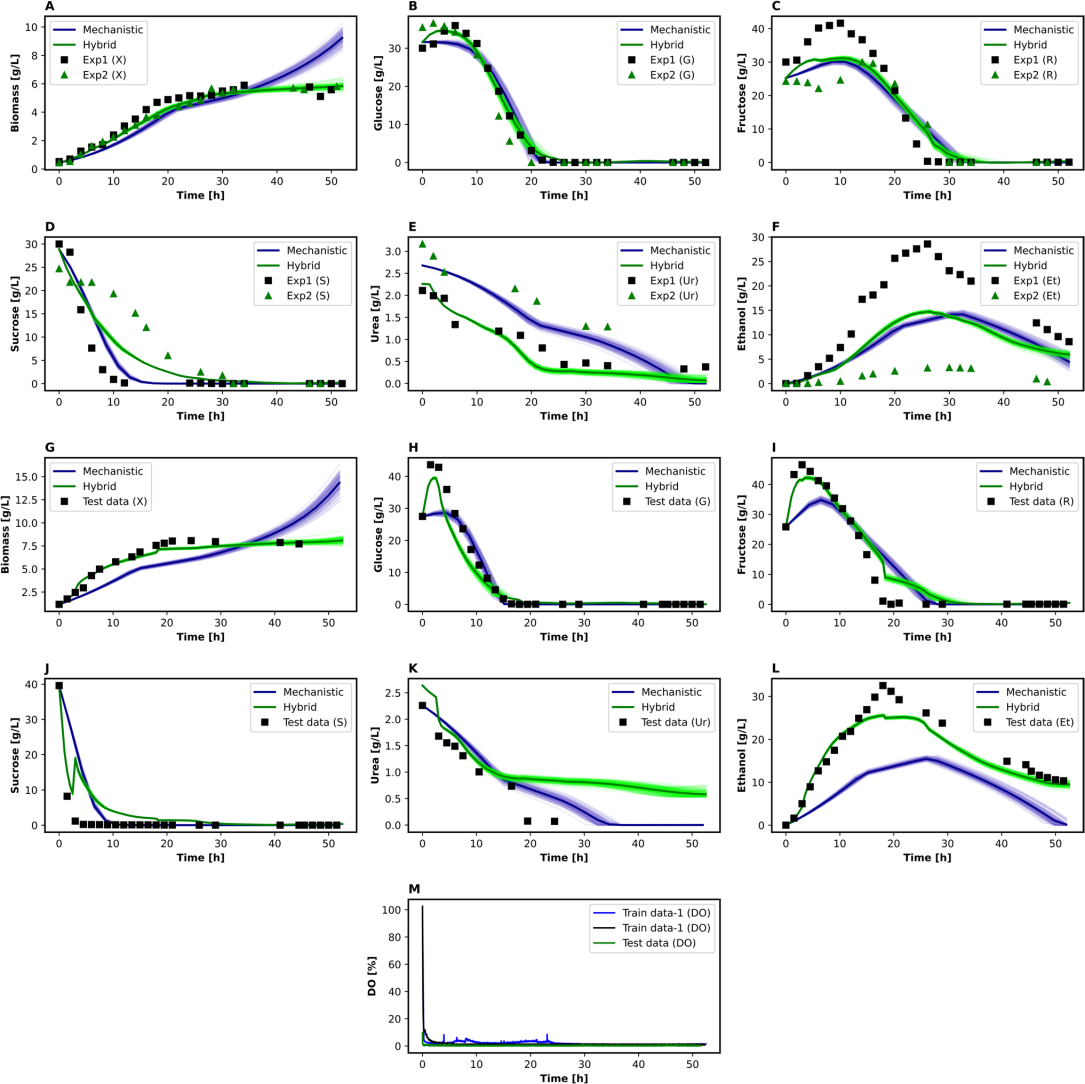
Concentration profiles of biological state variables predicted by the hybrid and mechanistic models during *S. cerevisiae* aerated batch fermentation. Panels **A**–**F** correspond to training data (Exp1 and Exp2), while panels **G**–**L** show predictions for test data. (**A, G**) Biomass, (**B**, **H**) Glucose, (**C**, **I**) Fructose, (**D**, **J**) sucrose, (**E**, **K**) Urea, and (**F**, **L**) Ethanol concentration. **M** Experimental dissolved oxygen concentration. Mechanistic model (Blue), hybrid model (Green). In plots **A**–**L**, ■ represents experimental data, while continuous lines represent model predictions. The blue and green shaded areas represent the error bands obtained from Monte Carlo simulations, which were performed using a 5% variation in the parameters for the mechanistic and hybrid models, respectively. In plot M, only experimental data are shown as a continuous line

Figure 3 compares the mechanistic (blue line) and hybrid (green line) models against experimental data for both the training (A–F) and test (G–L) data sets, considering ± 5% parameter uncertainty, while panel M depicts the measured dissolved oxygen concentration. The model’s stable response to these variations illustrates its robustness to parameter fluctuations, indicating that parameter changes within this interval exert minimal influence on predictive accuracy [84]. Overall, a coherent phenomenological alignment emerges, as each variable’s profile follows distinct kinetic patterns throughout the culture.

In batch-aerated yeast fermentations, two principal phases typically appear in the biomass concentration profile. During the initial phase, the specific growth rate is high owing to the abundance of simple sugars [71]. Under conditions of abundant sugar and oxygen, yeast rapidly metabolizes the available sugars and produces ethanol as a competitive strategy [85]. Under aerobic conditions, yeast can subsequently use the accumulated ethanol as a carbon source [85]. Thus, once simple sugars are depleted, biomass growth continues at a lower specific growth rate, driven by ethanol consumption, which defines the second phase [7, 71].

This transition in substrate utilization, known as the diauxic shift [86], is depicted in Fig. 3A/G, where the two main growth phases are both captured by the model. Nevertheless, after this second phase, the mechanistic model overestimates biomass concentration, whereas experimental data suggest a further drop in growth rate. This discrepancy may be explained by cell death, a factor currently omitted from the model. Accounting for cell death would increase the already intricate parameter set for mixed-sugar kinetics. However, our findings imply that accurately reproducing this phase could require an additional term, such as the logistic approach proposed by Ibáñez et al. [40], or a simpler Monod-type death rate expression.

*S. cerevisiae* secretes extracellular invertases that hydrolyze sucrose into glucose and fructose, enabling yeast assimilation of these simpler sugars [59, 87]. This enzymatic activity explains the rapid sucrose depletion initially observed. The sugar-consumption profile indicated that sucrose was hydrolyzed even in the presence of ≥ 10 g L⁻^1^ glucose. Although the inoculum was pre-grown in YMG, industrial baker’s strains typically retain basal extracellular invertase activity under glucose-repressing conditions [88]. Moreover, Olsson et al. (1997) [89] showed that naturally occurring lesions in the MIG repression system—common in bakery lineages—permit rapid derepression of SUC2 as soon as sucrose is encountered. Together, basal invertase carry-over and attenuated MIG-mediated catabolite repression explain the immediate sucrose hydrolysis observed.

During early cultivation, the hydrolysis rate outpaces the cellular uptake rate, causing transient accumulation of glucose and fructose, which are nonetheless being consumed. Once sucrose is depleted, glucose and fructose concentrations drop, dictated entirely by yeast uptake. They do so at distinct rates because of differing transporter affinities and regulatory mechanisms [90, 91]. Since glucose is typically the preferred carbon source, it suppresses the metabolism of other fermentable sugars, delaying fructose uptake [10]. Consequently, fructose consumption often accelerates only after glucose is depleted [92], which complicates modeling using simple mechanistic frameworks.

Although the mechanistic model reproduces the overall trends of mixed sugar consumption, discrepancies persist between predicted and observed concentrations (Fig. 3B/D, H/J). Such differences may stem from the complexities inherent in modeling mixed-sugar uptake rate, but they could also result from significant variations in the sucrose, fructose, and ethanol profiles between the training sets (Fig. 3C, D, F). For instance, the sucrose trajectory in “Exp 2” diverges markedly from both the other training run and the validation set. While different *Saccharomyces cerevisiae* strains can exhibit varying levels of invertase activity [93], with some strains showing reduced activity leading to delayed sucrose hydrolysis and reduced ethanol production, this explanation is unlikely in our case. Both experiments used the same strain and inoculum preparation protocol, minimizing biological variability. In addition, given the consistent UHPLC calibration and measurement procedures, analytical errors can be ruled out.

The observed variability appears to be associated with the combined influence of urea concentration and oxygen limitation. Urea may influence invertase expression and accelerate sucrose hydrolysis [87, 94] yet may also adversely affect yeast viability [95]. While low urea levels generally pose no adverse effect on yeast metabolism, changes in urea concentration across experiments might underlie these distinct sucrose profiles. In addition, small differences in oxygen availability may have occurred, even though operational conditions were nominally identical. Such discrepancies could arise from factors, such as impeller positioning, variability in the rotameter-regulated airflow, or partial clogging of the sparger. These subtle differences could result in differing degrees of oxygen limitation between experiments. Even when both cultures are under oxygen limitation, one culture may experience a more severe limitation, leading to divergent metabolic responses, particularly in ethanol production [96]. It is worth emphasizing that the current kinetic model does not explicitly account for oxygen availability, which may explain its inability to capture the observed differences. Thus, the variability is not merely experimental noise, but rather reflects intrinsic process uncertainty or a limitation of the mechanistic modeling approach, an aspect that is particularly relevant when compared to the performance of a hybrid model, and could also be addressed by further studies that include oxygen dynamics.

These mentioned factors together help explain the variability observed in the ethanol profiles between experiments (Fig. 3F/L). Despite these fluctuations, the mechanistic model effectively captured the main ethanol trend: an initial accumulation phase followed by a decline once all sugars were depleted. The rise in ethanol concentration reflects the Crabtree effect, whereby *S. cerevisiae* produces ethanol even under aerobic conditions when glucose is abundant [97]. This catabolic repression shifts yeast metabolism toward fermentation at the expense of respiration [98, 99]. A recent study associated this behavior with mitochondrial repression [100], yet the phenomenon’s complexity remains only partly understood, posing challenges for simple mechanistic frameworks. Overall, these findings highlight the inherent complexity of yeast metabolism and emphasize the need for refined modeling approaches to capture such multifaceted physiological responses.

Regarding the predicted urea concentration profile (Fig. 3E/K), the model successfully captures the initial rapid uptake rate, followed by shifts in uptake rate once glucose is depleted and again after all sugars are consumed. Nevertheless, some discrepancies arise in the magnitude and trend of the uptake rate. Under the tested conditions, urea transport involves both active mechanisms and facilitated diffusion [3], making it more complex to model than ammonium, which may follow simpler pathways [101]. Urea remains a cost-effective nitrogen source in large-scale *S. cerevisiae* processes, influencing growth, ethanol production, and parameters such as pH [3]. These considerations underscore the importance of accurately representing urea metabolism, rendering it a notable challenge for mechanistic modeling.

### Parameter reliability and identifiability of model structure via pre–post-regression analysis

Batch culture enables the identification of complex kinetic phenomena; however, experimental data collected in this mode often suffer from a lack of information, particularly regarding the half-saturation coefficients in Monod-type models [102]. This limitation naturally influences the PRA. Another factor that influences PRA is the sampling time. In this case, data were collected at varying intervals, with specific timepoints ranging from 0.5 to 3 h between experiments. In general, sampling occurred every 1.5–3 h, extending up to nearly 30 h of culture, though this varied across experiments. Then, fewer samples were taken as sugars were depleted and yeast growth slowed, sustained by ethanol consumption. To avoid bias in the relevance analysis due to insufficient experimental points, the PRA was applied to the data sets generated from the experimental data, as described in “Data Preparation and Preprocessing”.

Table 3 summarizes the PRA outcomes for the mechanistic model, revealing that 14 of 22 parameters were essential—each statistically significant (*t* value > 2), exhibiting meaningful sensitivities across model outputs and clear identifiability without notable correlations. Most estimated values remained close to their initial guesses and the literature-based bounds in Table 2, reflecting consistency with prior knowledge and confirming the effectiveness of the calibration strategy. These characteristics are crucial, because they ensure that the parameters can be reliably estimated from experimental data, thereby accurately representing the underlying biochemical and physiological processes. Moreover, this robust alignment not only preserves biological plausibility but also reinforces the model’s capacity to capture key processes—such as urea consumption, biomass growth on glucose, sucrose hydrolysis, and the dual role of ethanol as both a by-product and an inhibitor—ultimately enhancing the model’s generalizability, reproducibility, and reliability for real-time process optimization, advanced control strategies, and decision-making in industrial bioprocesses.

**Table 3:** Summary of results in parameter estimation and analysis. Parameters in white background are significant, and otherwise non-significant. “No Id.” refers to non-identifiable parameters

Parameter	Units	Initial value	Estimate	%${\sigma }_{\theta }$	Lower bound	Upper bound
${\mu }_{maxEt}$	g/(gh)	0.090	0.007	No Id	–	–
${\mu }_{max,G}$	g/(gh)	0.098	0.149	1.83%	0.146	0.152
${\mu }_{max,R}$	g/(gh)	0.096	0.011	No Id	–	–
${\mu }_{max,R,postG}$	g/(gh)	0.265	0.047	No Id	–	–
${m}_{G}$	g/(gh)	0.047	0.037	No Id	–	–
${m}_{R}$	g/(gh)	0.057	0.128	7.42%	0.118	0.137
$\alpha$	g/(gh)	11.815	7.645	13.30%	6.629	8.662
${K}_{in{h}_{AF}}$	g/L	0.186	0.203	87.98%	0.024	0.382
${K}_{Et}$	g/L	0.108	0.092	No Id	–	–
${K}_{i,Et}$	g/L	12.836	7.920	6.01%	7.445	8.396
${K}_{G}$	g/L	0.057	0.035	27.17%	0.026	0.045
${K}_{R}$	g/L	10.548	1.666	No Id	–	–
${K}_{R, post G}$	g/L	0.001	6.768e–09	No Id	–	–
${K}_{S}$	g/L	38.983	29.935	19.68%	24.043	35.826
${Y}_\frac{AF}{S}^{dis}$	g/g	0.563	0.520	7.42%	0.481	0.559
${Y}_\frac{Et}{G}$	g/g	0.891	0.451	3.82%	0.434	0.468
${Y}_\frac{Et}{R}$	g/g	0.900	0.740	9.48%	0.670	0.810
${Y}_\frac{Et}{X}$	g/g	5.409	3.126	0.69%	3.105	3.148
${Y}_{\frac{Et}{X},c}$	g/g	5.603	10.907	No Id	–	–
${Y}_\frac{G}{X}$	g/g	5.000	5.417	No Id	–	–
${Y}_\frac{R}{X}$	g/g	3.000	7.488	No Id	–	–
${Y}_\frac{X}{Ur}$	g/g	3.590	2.794	1.26%	2.759	2.829

Table 3 PRA outcomes indicate that most parameters associated with fructose metabolism—particularly those related to fructose uptake—are non-significant. The complex dynamics governing the utilization of glucose and fructose are underscored by the fact that, although both sugars share the same plasma membrane transport system, the transporters exhibit a higher affinity for glucose than for fructose [10]. Furthermore, ethanol exerts an inhibitory effect [61], which is more pronounced on fructose utilization compared to glucose utilization [103]. Limited data variability may also have obscured distinct behaviors that would otherwise be revealed by more informative parameters.

Among the fructose-related parameters, only ${m}_{R}$ and ${Y}_\frac{Et}{R}$ (both linked to fructose consumption) and ${Y}_\frac{AF}{S}^{dis}$ (associated with fructose production and partially related to glucose metabolism) were found to be significant. These results suggest that ${m}_{R}$, ${Y}_\frac{Et}{R}$, and ${Y}_\frac{AF}{S}^{dis}$ are sufficient to describe the fructose concentration profile, while the other fructose parameters do not substantially contribute to this profile or other state variables, such as biomass. Consequently, predictions of biomass concentration are predominantly influenced by glucose kinetics, which is plausible, because each parameter contributes uniformly to total biomass formation. Deviations in the bounds of fructose parameters—likely resulting from the substitution with glucose-based parameters and the application of a dynamic bounds approach, as described in “Parameter Estimation via Global Optimization”—may further skew the model toward glucose-associated behavior.

There is a tendency for all yield parameters to exhibit confidence intervals below ± 9.8%, whereas inhibition or saturation constants display higher uncertainties, ranging from ± 6.1% to ± 27.17%, and reaching 87.98% in the most extreme case (${K}_{in{h}_{AF}}$). Although ${K}_{in{h}_{AF}}$ and ${K}_{G}$ had near-zero sensitivity (data not shown), they remain phenomenologically necessary for describing ethanol consumption and glucose uptake transitions, thus warranting inclusion in the model.

Only two of the 14 significant parameters reported in Table 3 were non-identifiable (${\mu }_{maxEt}$ and ${Y}_{\frac{Et}{X},c}$), both associated with *S. cerevisiae*’s ethanol consumption. Reevaluating how ethanol uptake kinetics integrate with biomass generation appears necessary when ethanol is the only carbon source. Currently, the ethanol uptake rate equation (Eq. 15) closely mirrors the biomass growth rate (Eq. 9) for depleted sugars, aside from the inclusion of ${Y}_{\frac{Et}{X},c}$. While this approach correctly reflects ethanol’s principal conversion to biomass, it complicates parameter identifiability. In contrast, the method proposed by Xu [104] uses a maximum ethanol consumption rate (${q}_{Et,c,max}$), effectively decoupling ethanol consumption from growth and potentially resolving identifiability issues. However, it may diverge from the core aim of the present model, which highlights the interplay between ethanol consumption and biomass formation.

### Hybrid modeling with LSTM neural networks for enhanced fermentation predictions

Figure 3 compares the mechanistic (blue line) and hybrid (green line) models against experimental data for both the training (A–F) and test (G–L) data sets, while panel M depicts the measured dissolved oxygen concentration. Below, we examine the performance of each model for the six key state variables—biomass, glucose, fructose, sucrose, urea, and ethanol—highlighting the distinct behaviors observed in the training and test data sets.

1. Biomass (Fig. 3A,  G): In the training data set (Fig. 3A), the hybrid model closely matches experimental biomass observations throughout the entire cultivation, including later timepoints, where the mechanistic model tends to overestimate growth. This improvement is also evident in the test data set (Fig. 3G), demonstrating the hybrid model’s robustness in capturing biomass accumulation even when new experimental conditions are introduced.
2. Glucose (Fig. 3B,  H): For both training and test runs, the mechanistic model underestimates glucose levels during the early stages when sucrose hydrolysis generates additional glucose. The hybrid model corrects this bias (Fig. 3B,  H) by leveraging residual information, yielding a closer alignment with the measured data.
3. Fructose (Fig. 3C, I): Like glucose, fructose concentrations are better captured by the hybrid approach in both data sets, especially during transitions between simple sugars. Figure 3C,  I shows that the mechanistic model lags behind the observed fructose trends, whereas the hybrid approach tracks them more accurately.
4. Sucrose (Fig. 3D, J): Within the training data set (Fig. 3D), the mechanistic model generally tracks sucrose depletion but fails to capture the initial decline rate in “Exp 2.” The hybrid model, however, significantly reduces this discrepancy. Likewise, under test conditions (Fig. 3J), the hybrid approach more accurately depicts sucrose dynamics, despite a noticeable discontinuity around the 2‑h mark. This jump results from how the neural network component processes time‐series inputs with a fixed window length of *p* = 5. After predicting the first five data points, the model shifts to a new sequence, creating an abrupt change in the plotted profile. Nonetheless, the hybrid model still represents the overall sucrose depletion trend more faithfully than the purely mechanistic alternative.
5. Urea (Fig. 3E, K): In the training data set (Fig. 3E), the hybrid model more closely follows urea depletion dynamics than the mechanistic model, particularly during later phases after sugar exhaustion. However, for the test data set (Fig. 3K), the hybrid model’s predictions diverge significantly from the experimental data beyond approximately 20 h, performing worse than the mechanistic model in that time window. This discrepancy likely arises, because the training data exhibit a specific urea uptake rate pattern past 20 h, which the hybrid approach reproduces but which does not adequately match the test data set’s actual profile.
6. Ethanol (Fig. 3F, L): Ethanol concentrations present some of the greatest variability between training experiments, posing a challenge for the mechanistic model. Nonetheless, the hybrid model better adjusts to these fluctuations (Fig. 3F,  L), thereby providing more reliable predictions in both data sets. This demonstrates the hybrid model’s superior ability to capture dynamic patterns that traditional mechanistic models fail to represent [105].

Overall, Figs. 3A–L shows that the hybrid model not only refines predictions for the training data but also generalizes effectively to unseen test data, achieving enhanced accuracy across biomass, sugars, urea, and ethanol. This improvement is further supported by the Root Mean Square Error (RMSE) analysis **(**Table 4**)**, where the hybrid approach reduces error by approximately 1.9‐fold in training and 2.0‐fold in testing compared to the mechanistic model. RMSE values were calculated by comparing experimental data points with each model’s predictions at corresponding timepoints, thereby quantifying the average prediction error. Consequently, the consistently lower RMSE values of the hybrid model indicate that combining a simple mechanistic core with data‐driven corrections effectively mitigates model bias, resulting in stronger predictive power for both training and testing scenarios.

**Table 4:** Estimated root mean square error (RMSE) for each variable compared with test data

	Models	Biomass (X)	Glucose (G)	Fructose (R)	Sucrose (S)	Urea (Ur)	Ethanol (Et)	Total
Train data	Mechanistic	1.27	2.55	2.93	2.87	0.42	1.69	1.95
	Hybrid	0.22	1.49	1.94	0.95	0.68	0.87	1.03
Test data	Mechanistic	2.27	4.69	7.50	7.27	0.72	11.14	5.60
	Hybrid	0.48	3.61	4.55	5.32	0.43	2.80	2.87

Furthermore, the previous results also highlight the generalization capabilities of hybrid modelling, in which a limited training set—conditioned by the restrained data acquisition for the number of evaluated variables—was able to account for differentiated variable dynamics that were not represented by the mechanistic model and not explicitly present in the training data. In this case, the behavior of glucose and fructose maximum concentrations in Fig. 3H, I shows that the data-driven approach was able to approximate the steep hydrolytic kinetics and monosaccharide accumulation during the first hours of the test experiment. Therefore, the predictive performance of the hybrid model supports the potential of employing hybrid frameworks as tools for reaching higher predictive capacity under data scarcity conditions, in comparison with purely mechanistic and data-driven models.

### General discussion

The accurate modeling of *S. cerevisiae* fermentation under mixed carbon sources and urea as a nitrogen source was successfully addressed through a hybrid modeling framework. This approach combined mechanistic modeling based on mass balances and substrate consumption kinetics with a data-driven neural network to capture complex metabolic behaviors, such as diauxic shifts, mixed sugar uptake, and ethanol production. The initial mechanistic model underwent robust global parameter estimation using grey wolf optimization (GWO), complemented by systematic pre- and post-regression analyses to guarantee parameter identifiability, sensitivity, and confidence intervals, enhancing the overall reliability of the model.

The hybrid model demonstrated superior predictive performance, significantly reducing the prediction errors compared to purely mechanistic modeling approaches. Specifically, the predictive error decreased by approximately a factor of two, providing a reliable representation of culture dynamics under diverse operational conditions. These findings align with recent studies, including those by Ibáñez et al. [40], where hybrid models demonstrated considerable improvements in predictive accuracy for fed-batch fermentations, particularly under complex high-cell-density and metabolic overflow conditions.

An important consideration in the presented hybrid approach is the incorporation of neural network architectures. Long–short-term memory (LSTM) neural networks were specifically advantageous for modeling time-dependent biological processes compared to other neural network architectures, such as feed-forward or convolutional neural networks [106, 107]. LSTM networks inherently capture temporal dependencies and sequence-related dynamics, effectively managing historical data points [108] essential for accurately modeling metabolic shifts and transitions, common in yeast fermentations [70]. Compared to feed-forward neural networks, which neglect temporal correlations, LSTM architectures provide superior accuracy in predicting dynamic behavior, making them especially valuable for bioprocess applications involving time-dependent substrate interactions and metabolic changes.

Future research should extend this integrated hybrid model by coupling the developed biological submodel with physicochemical and reactor-scale dynamics, thus providing comprehensive coverage from cellular metabolism to reactor-scale phenomena. A significant advantage of employing hybrid models equipped with LSTM networks is their ability to facilitate advanced real-time control strategies and optimize feeding policies and nutrient supply in bioprocess operations. This could enable enhanced predictability, operational robustness, and resource efficiency. In addition, future research could explore adaptive LSTM structures that dynamically adjust to process variability, further strengthening the model’s role in real-time optimization and precise bioprocess control.

## Conclusions

This study presents a groundbreaking hybrid modeling framework for *Saccharomyces cerevisiae* aerated batch fermentations, seamlessly integrating a biologically grounded mechanistic core with data-driven residual corrections, significantly boosting predictive performance while maintaining interpretability. The mechanistic model, developed through rigorous pre- and post-regression analysis, accurately represents key biological processes using identifiable parameters and effectively accounts for critical phenomena, such as mixed-sugar co-utilization and ethanol metabolism. Leveraging this robust foundation, the hybrid model excels in fitting experimental data by learning systematic deviations—residuals—between mechanistic predictions and actual observations, enabling accurate representation of complex dynamic behaviors, such as the diauxic shift and non-linear sugar utilization kinetics. In comparison with the mechanistic model alone, the hybrid approach achieved an impressive average 1.9-fold reduction in prediction error during training and a 2.0-fold reduction during testing across biomass, substrate, nitrogen, and product concentrations. Notably, this outstanding performance is achieved without an overly complex kinetic expression that could compromise parameter identifiability. These results underscore the immense potential of hybrid models as practical, scalable solutions for navigating the inherent complexity of yeast fermentations and propelling robust, data-integrated strategies for process optimization and digital bioprocess control in industrial biotechnology.

## Supplementary Information

Below is the link to the electronic supplementary material.

Supplementary file1 (DOCX 392 KB)

## Data Availability

No data sets were generated or analysed during the current study.

## List of symbols

$\mu Et$: Specific rate of ethanol generation (*h*^*−1*^)
$\mu G$: Specific rate of biomass formation due to glucose (*h*^*−1*^)
${\mu }_{max,Et}$: Maximum specific rate of biomass formation due to Ethanol (*h*^*−1*^)
${\mu }_{max,G}$: Maximum specific rate of biomass formation due to glucose (*h*^*−1*^)
${\mu }_{max,R}$: Maximum specific rate of biomass formation due to fructose (*h*^*−1*^)
${\mu }_{R}$: Specific rate of biomass formation due to fructose (*h*^*−1*^)
${\mu }_{max,postG}$: Maximum specific rate of biomass formation due to fructose, once glucose is depleted (*h*^*−1*^)
$\mu x$: Specific cell growth rate (*h*^*−1*^)
$rcEt$: Ethanol uptake rate (*g L*^*−*^^*1*^* h*^*−*^^*1*^)
$rEt$: Ethanol production rate (*g L*^*−*^^*1*^* h*^*−*^^*1*^)
$rG$: Glucose uptake rate (*g L*^*−*^^*1*^* h*^*−*^^*1*^)
$rgG$: Glucose production rate (*g L*^*−*^^*1*^* h*^*−*^^*1*^)
$rgR$: Fructose production rate (*g L*^*−*^^*1*^* h*^*−*^^*1*^)
$rR$: Fructose uptake rate (*g L*^*−*^^*1*^* h*^*−*^^*1*^)
$rS$: Sucrose uptake rate (*g L*^*−*^^*1*^* h*^*−*^^*1*^)
$rUr$: Urea uptake rate (*g L*^*−*^^*1*^* h*^*−*^^*1*^)
$rx$: Biomass growth rate (*g L*^*−*^^*1*^* h*^*−*^^*1*^)
$YEt/G$: Ethanol/glucose yield (*g g*^*−*^^*1*^)
$YEt/X$: Ethanol/biomass yield (*g g*^*−*^^*1*^)
$YG/S$: Glucose/sucrose yield (*g g*^*−*^^*1*^)
$YQ/G,ox$: Heat/glucose yield under oxidation state (*J kg*^*−*^^*1*^)
$YQ/R,ox$: Heat/fructose yield under oxidation state (*Jkg*^*−*^^*1*^)
$YR/S$: Fructose/sucrose yield (*g g*^*−*^^*1*^)
$YX/G,ox$: Biomass/glucose yield under oxidation state (*g g*^*−*^^*1*^)
$YX/R,ox$: Biomass/fructose yield under oxidation state (*gg*^*−*^^*1*^)
$\alpha S$: Sucrose hydrolysis parameter (gS gX^*−*^^*1*^* h*^*−*^^*1*^)
$KG$: Half-saturation coefficient for glucose (*gL*^*−*^^*1*^)
$KR$: Half-saturation coefficient for fructose (*gL*^*−*^^*1*^)
$KEt$: Half-saturation coefficient for ethanol (*gL*^*−*^^*1*^)
$KInhEt$: Half-saturation coefficient for ethanol inhibition (*gL*^*−*^^*1*^)
${K}_{Et,AF}^{inh}$: Half-saturation coefficient for ethanol inhibition under fermentative metabolism (*gL*^*−*^^*1*^)
$\epsilon$: Leaky ReLu stabilization term for normalization (dimensionless)
$\gamma$: Leaky ReLu leak factor (dimensionless)
