# Supplementary material for: Advancing hybrid modeling of Saccharomyces cerevisiae fermentation with mixed carbon sources and urea in a mini-stirred tank reactor
Source: Bioprocess Biosyst Eng. 2025 Aug 23;48(11):1919–37. doi: 10.1007/s00449-025-03222-5 (PMC12511178; doi:10.1007/s00449-025-03222-5)
Supplement: Supplementary file 1 — Supplementary file1 (DOCX 392 KB) [file 449_2025_3222_MOESM1_ESM.docx]

**Supporting Information to:**

**Advancing Hybrid Modeling of Saccharomyces cerevisiae Fermentation with Mixed Carbon Sources and Urea in a Mini-Stirred Tank Reactor**

Valencia-Velásquez, Jhonatan **^a^**; Yaker-Moreno, Hector Andres **^a^**; Martínez Guerrero,
Alejandro **^a^**; Ibáñez-Espinel, Francisco **^c^**; Pérez-Correa, José Ricardo **^d^**; Caicedo-Ortega, Nelson H. **^a, b^**

*Nelson H. Caicedo-Ortega
nhcaicedo@icesi.edu.co*

nhcaicedo@icesi.edu.co

**a** *Departamento de Ciencias Biológicas, Bioprocesos y Biotecnología, Facultad de Ingeniería, Diseño y Ciencias aplicadas, Universidad ICESI, Cali*

**b** *Centro BioInc. Universidad ICESI, Cali*

**c** *Advanced Process Control & Data Sciences, Natural Resources, SGS Chile, Puerto Madero # 130, Pudahuel, Santiago, Chile; Teléfono:+ 562 2898 9500.*

**d** *Departamento de Ingeniería Química y de Bioprocesos, Facultad de Ingeniería, Pontificia Universidad Católica de Chile, Casilla 306 Correo 22, Santiago, Chile; Teléfono: +562 354 49 27; Fax: +562 354 58 03.*

**S1. Carbon Sources Analysis**

The analysis of carbon sources was carried out by quantifying the concentrations of sucrose, glucose, fructose, and ethanol throughout the fermentation process using ultra-high-performance liquid chromatography (UHPLC). At each sampling point, 2 mL of culture was collected. The supernatant was obtained by centrifugation and stored at −4 °C until analysis.

Quantification was performed using a UHPLC system (UltiMate 3000, Thermo Fisher Scientific, USA) equipped with a refractive index detector (RID) and an Aminex® HPX-87H column (300 × 7.8 mm, 10 μm, Thermo Fisher). The column was operated at 35 °C, using 5 mM sulfuric acid as the mobile phase at a flow rate of 0.6 mL min⁻¹. Calibration curves were prepared using standard solutions of sucrose, glucose, fructose, and ethanol at concentrations ranging from 0.1 to 8.0 g L⁻¹.

Table S1 presents the calibration data provided by the analytical laboratory to evaluate the performance of the UHPLC column. Representative UHPLC chromatograms of standard solutions used for the calibration of carbon sources and ethanol are shown in Figures S1–S5. The chromatographic peaks correspond to sucrose, glucose, fructose, and ethanol, as indicated in each figure.

**Table S1.** Calibration data for UHPLC quantification of sucrose, glucose, fructose, and ethanol

| **Code** | **Calibration level** | **Component** | **Calibration curve R-Square** | **Calibration curve X Value** | **Calibration curve Y Value** | **Calibration curve Area (µRIU*min)** | **Calibration curve Height (µRIU)** |
| --- | --- | --- | --- | --- | --- | --- | --- |
| STD1 | 1 | Sucrose | 0,9999 | 0,7964 | 3,3030 | 3,3030 | 10,6868 |
| STD2 | 2 |  |  | 2,7874 | 11,7619 | 11,7619 | 38,2880 |
| STD3 | 3 |  |  | 9,9550 | 41,7111 | 41,7111 | 135,3682 |
| STD4 | 4 |  |  | 23,8920 | 101,0807 | 101,0807 | 324,7325 |
| STD5 | 5 |  |  | 39,8200 | 165,8221 | 165,8221 | 522,4664 |
| STD1 | 1 | Glucose | 0,9801 | 0,8019 | 3,5825 | 3,5825 | 10,2100 |
| STD2 | 2 |  |  | 2,0048 | 12,7801 | 12,7801 | 36,5115 |
| STD3 | 3 |  |  | 10,0240 | 45,2973 | 45,2973 | 128,8833 |
| STD4 | 4 |  |  | 30,0720 | 109,9277 | 109,9277 | 311,2870 |
| STD5 | 5 |  |  | 40,0960 | 180,3995 | 180,3995 | 500,4557 |
| STD1 | 1 | Fructose | 0,9802 | 0,7986 | 3,6132 | 3,6132 | 10,5514 |
| STD2 | 2 |  |  | 1,9966 | 12,8821 | 12,8821 | 37,8308 |
| STD3 | 3 |  |  | 9,9830 | 45,6851 | 45,6851 | 133,4833 |
| STD4 | 4 |  |  | 29,9490 | 110,8663 | 110,8663 | 322,1684 |
| STD5 | 5 |  |  | 39,9320 | 181,8251 | 181,8251 | 517,1586 |
| STD1 | 1 | Ethanol | 0,9812 | 0,2017 | 0,3968 | 0,3968 | 0,8109 |
| STD2 | 2 |  |  | 0,5042 | 1,4141 | 1,4141 | 2,8750 |
| STD3 | 3 |  |  | 2,5210 | 5,0147 | 5,0147 | 10,2148 |
| STD4 | 4 |  |  | 7,5630 | 12,1964 | 12,1964 | 24,5877 |
| STD5 | 5 |  |  | 10,0840 | 19,8848 | 19,8848 | 39,5341 |

**S2. Urea concentration quantification**

Urea concentration was determined using the Megazyme Urea/Ammonia (Rapid) Assay Kit, based on a standard enzymatic method involving two sequential reactions. First, urea is hydrolyzed by urease, and subsequently, the released ammonia reacts with 2-oxoglutarate in the presence of glutamate dehydrogenase, consuming NADPH. The decrease in NADPH is stoichiometrically proportional to the urea concentration and is monitored by measuring the reduction in absorbance at 340 nm [1].

For each time point, 2 mL of culture was collected and centrifuged to obtain the supernatant, which was then stored at −4 °C until analysis. Given that the supernatant was clear or slightly colored, the procedure for liquid samples was followed according to the kit’s assay procedure [1]. Aliquots of 0.1 mL were used per assay, and absorbance was measured using a UV-Vis spectrophotometer (Thermo Scientific Genesys 15, model 480-300000). Calculations were performed using Megazyme Mega-Calc^TM^ provided in www.megazyme.com/urea-ammonia-assay-kit (prod-docs.megazyme.com/documents/Data_Calculator/K-URAMR_CALC.xlsx).

**
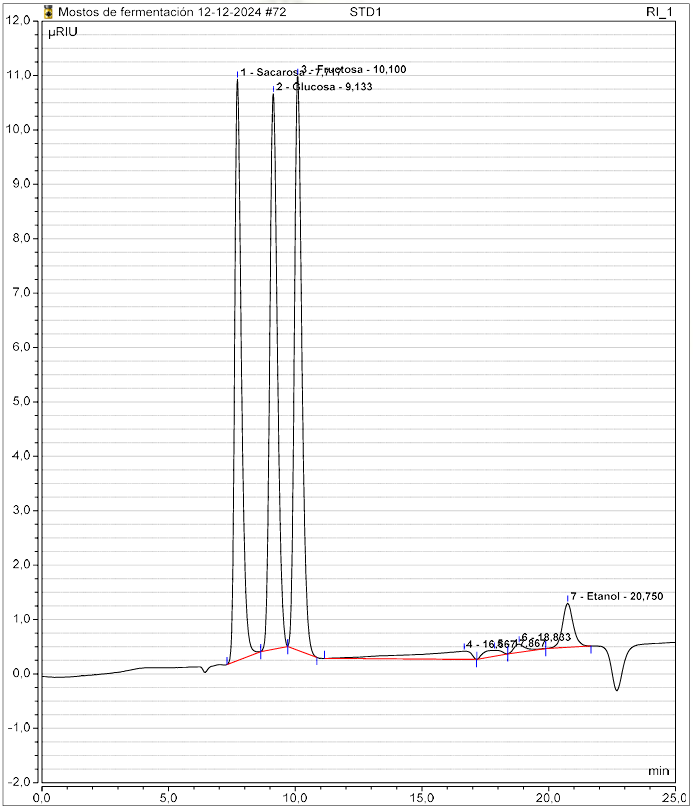
**

**Figure S1.** Chromatogram of Standard Sample 1.


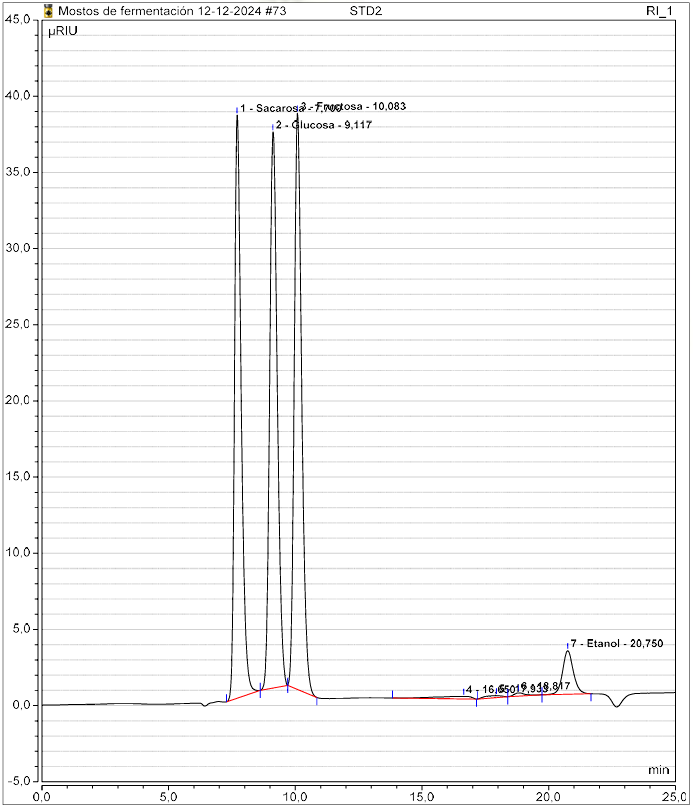


**Figure S2.** Chromatogram of Standard Sample 2.

**
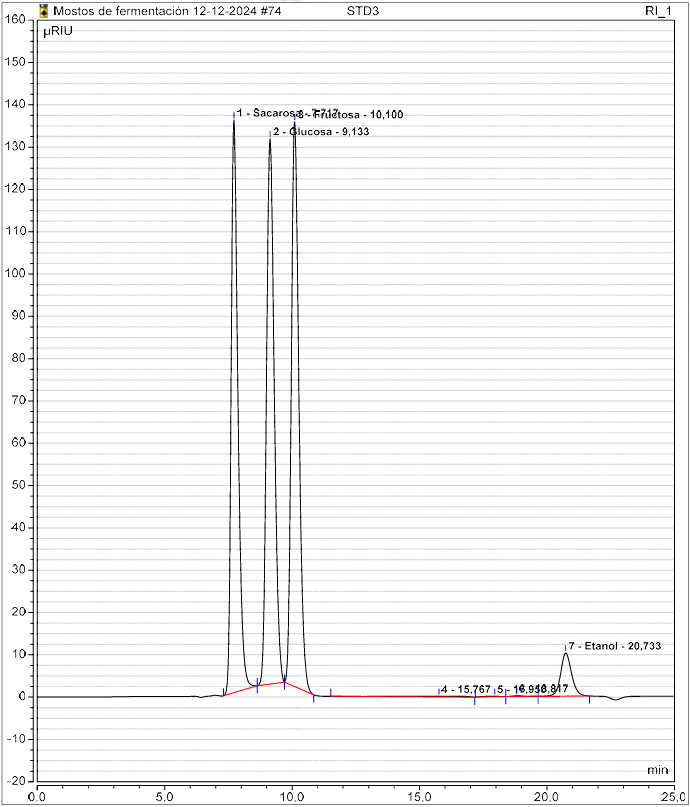
**

**Figure S3.** Chromatogram of Standard Sample 3.

**
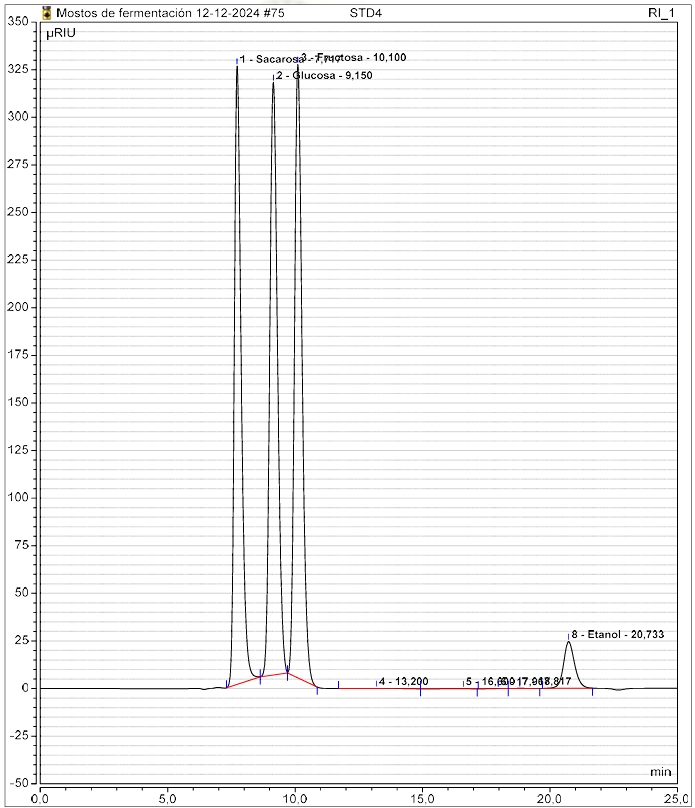
**

**Figure S4.** Chromatogram of Standard Sample 4.


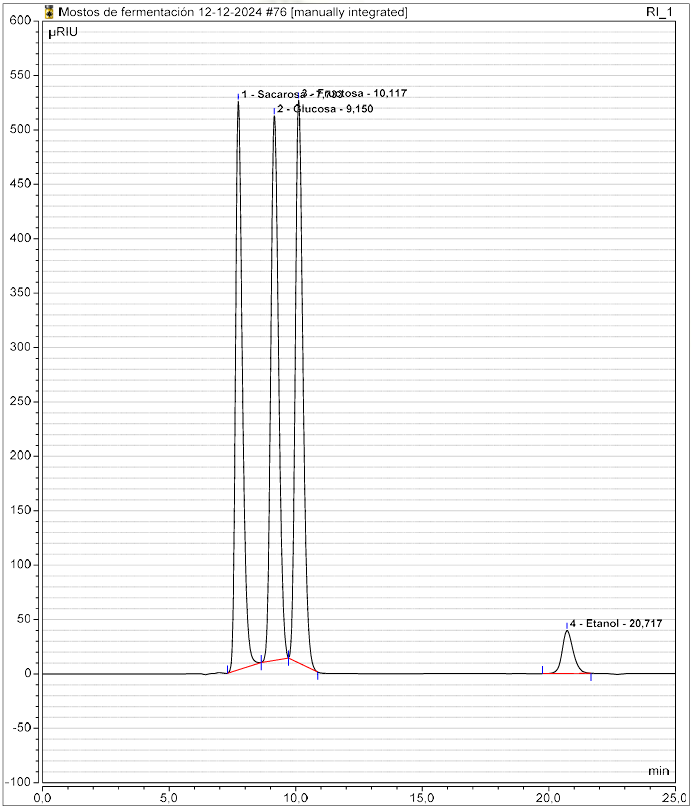


**Figure S5.** Chromatogram of Standard Sample 5.

**References**

[1] Megazyme, *Urea/Ammonia (Rapid) Assay Procedure (K-URAMR)*, Megazyme International, Bray, Ireland. [Online]. Available: <https://prod-docs.megazyme.com/documents/Assay_Protocol/K-URAMR_DATA.pdf> [Accessed: Jul. 2, 2025].
